# Changes in Tobacco Use Patterns among Veterans in San Diego during the Recent Peak of the COVID-19 Pandemic

**DOI:** 10.3390/ijerph182211923

**Published:** 2021-11-13

**Authors:** Javad J. Fatollahi, Sean Bentley, Neal Doran, Arthur L. Brody

**Affiliations:** 1 Department of Psychiatry, University of California San Diego, La Jolla, CA 92093, USA; nmdoran@health.ucsd.edu (N.D.); abrody@health.ucsd.edu (A.L.B.); 2 School of Medicine, University of California San Diego, La Jolla, CA 92093, USA; ssbentle@health.ucsd.edu; 3 Mental Health Care Line, VA San Diego Healthcare System, San Diego, CA 92161, USA

**Keywords:** tobacco use disorder, tobacco dependence, cigarette smoking, veteran, COVID-19 pandemic, substance use disorder, serious mental illness

## Abstract

The prevalence of tobacco use increases in times of stress; however, during the initial stage of the COVID-19 pandemic, tobacco use rates stayed the same in most populations. Previous work focused on the initial months of the pandemic, while this study examined the changes in tobacco use during a later peak period of the pandemic. We used data from 61,852 visits to the VA San Diego Healthcare System from November 2019 to February 2021, divided into pre-, early, and peak pandemic periods. Multinomial logistic regression was used to test whether the odds of being a daily or non-daily tobacco user varied over time, by demographic group, or with the presence of specific psychiatric diagnoses. Younger Veterans had a greater reduction in the prevalence of non-daily tobacco use between the early and peak periods, while older Veterans had a rise in daily use from pre- to the early pandemic, which returned to baseline during the peak. Individuals with substance use disorder and serious mental illness diagnoses were more likely to report tobacco use, but psychiatric diagnoses did not predict change over time. These findings demonstrate factors that potentially contribute to changes in tobacco use during a public health crisis and may help guide future targeted cessation efforts.

## 1. Introduction

Several cross-sectional studies have shown that perceived stress leads to increased rates of cigarette smoking [1,2]. Multiple reports have also shown that the prevalence of tobacco use increased after anthropogenic and natural disasters, such as the 11 September 2001 attacks [3], Hurricane Katrina [4], and the Japanese tsunami in 2011 [5], where rates of tobacco use in survivors were near double the national averages. The COVID-19 pandemic is the most recent global disaster, with over 250 million infections and 5 million deaths worldwide as of 9 November 2021 [6]. The COVID-19 pandemic reached San Diego County in Southern California in mid-March 2020 and led to the county’s first coronavirus-related shutdown. According to the San Diego County Health and Human Services Agency (HHSA), there have been a total of 335,302 positive COVID-19 cases and 3908 deaths since 14 February 2020 (data through 29 August 2021) [7]. Isolation and insecurity were an early byproduct of the pandemic due to government and state-mandated lockdowns and quarantines. These social restrictions and concern about well-being have manifested as an overwhelming level of stress in many individuals throughout the world. A recent study demonstrated that sociodemographic predictors of greater COVID-19-related distress included being female, unemployed, and divorced [8].

During the initial stages of the COVID-19 pandemic, tobacco use rates appeared to have stayed the same overall, despite the increased level of stress, due to the majority of users reporting the same amount of tobacco use, with smaller subgroups reporting either increased or decreased use [9,10,11,12,13,14,15]. A survey conducted in the United States in April 2020 showed that of the 366 subjects surveyed, 41.4% had not changed their tobacco use habits, 28.3% had decreased their use, and 30.3% had increased their use since learning of COVID-19 [16]. Another survey conducted around the same period had a total of 6800 respondents from the United States, the United Kingdom, Italy, India, and South Africa, and yielded similar results [17]. Some common reasons for increased tobacco use included boredom while quarantined, more time at home, and increased stress. The most common reasons for decreased tobacco use were perceptions of greater risks of both becoming infected and suffering severe complications due to COVID-19, along with more time spent around non-users [18]. While the majority of studies suggest that tobacco use rates stayed the same during the initial stages of the pandemic, some studies did show that overall rates either increased [19,20,21] or decreased [22,23,24,25]. Taken together, these studies demonstrate that overall tobacco use rates remained largely unchanged during the early stages of the pandemic, at least in the populations cited above.

In California, a later peak stage of COVID-19 emerged in November 2020, with new cases climbing above 10,000 per day for a sustained period of time [26]. This later stage can be marked by California Governor Gavin Newsom’s announcement of more stringent restrictions on 16 November 2020, in which he ordered almost all non-essential businesses to close and mandatory mask use whenever Californians were outside their homes. Daily cases continued to rise during this time period, with media and public concern at an all-time high over the winter season, which saw the Southern California Intensive Care Unit capacity drop to 0% in December 2020 [27] and the daily case rates peaking in January 2021 at over 60,000, a greater than ten-fold increase from earlier in the pandemic [26]. This wave receded in February 2021, with cases in California dropping below 5000 per day for the first time since October 2020 [26]. While previous studies have investigated the effect of the early COVID-19 pandemic on tobacco use rates, we are not aware of studies examining rates of tobacco use over various phases of the pandemic, especially during this wave of cases that occurred during the fall and winter of 2020–2021.

In recent years, electronic medical records (EMR) have become the predominant method of record keeping, and both facilitators and barriers to adoption having been identified [28]. The EMRs contain data collected during routine healthcare visits [29], and many recent studies have utilized EMR information to examine the effects of the pandemic on patients screening positive for health-related issues [30,31,32]. The study presented here used EMR-based data to examine the prevalence of tobacco use over three specific time periods between November 2019 to February 2021, before and during which the COVID-19 pandemic had a significant exacerbation in California and the rest of the United States [26]. We sought to test whether the overall tobacco use rates changed from before to during the peak part of the pandemic and whether changes were moderated by demographics or psychiatric diagnoses. Based on the previous studies cited above, we expected to see a worsening of overall rates of tobacco use during the early and peak periods of the COVID-19 pandemic, and that certain demographic subgroups might have altered patterns of tobacco use behavior. For example, prior studies showed that decreased tobacco use was more apparent in younger age groups [24] and among women [25].

## 2. Materials and Methods

### 2.1. Data Extraction and Variables Used

VA San Diego Healthcare System (VASDHS) providers are required to conduct annual tobacco use screenings, which consist of a brief series of questions about current and past tobacco use. We used data from 61,852 visits to VA providers from November 2019 to February 2021, gathered from electronic medical records stored in the VA’s Corporate Data Warehouse. This research study was approved by the local Institutional Review Board for the VA San Diego Healthcare System. All the visits included in the study incorporated the tobacco use screening as part of the encounter. Current tobacco use status was the primary outcome measure of the study and was assessed using the question “Do you smoke cigarettes or use tobacco every day, some days, or not at all?” Providers recorded patient responses in the EMR, attaching digital objects called health factors to the specific visit record, and allowing the generation of lists of visits meeting specific criteria. For this project, we generated lists of visits at which patients reported current tobacco use status (daily user, non-daily user, or non-user). Our primary outcomes of interest were the prevalence of daily tobacco use and the prevalence of non-daily tobacco use. Other variables of interest consisted of demographic variables and psychiatric/substance use diagnoses. We coded each visit in terms of whether it occurred during the pre-COVID (1 November 2019–18 March 2020), early COVID (19 March 2020–15 November 2020), or peak COVID (16 November 2020–28 February 2021) periods. The “early” and “peak” COVID periods were defined based on the events and daily case rates cited above. Because the tobacco assessment was due annually, there were a relatively small number of instances of multiple visits for the same individual patient. Within time periods, if duplicate visits for any unique patient occurred, only the first visit was retained (n = 254 records were excluded for this reason). There were 10,856 patients who had visits and tobacco use status data in two separate periods. Thus, the 61,852 total visits included in the analyses represented 50,966 unique patients.

Demographic information (age at the time of the visit, gender, and racial/ethnic identity) and psychiatric/substance use diagnoses were also included in the analysis. VA medical records include demographics and a problem list, which identifies active health problems for each patient by ICD-10 code. Diagnostic predictors were adapted from VA Mental Health Strategic Analytics for Improvement and Learning (SAIL) metrics [33]. More specifically, we created predictors indicating whether or not patients’ problem lists included diagnoses in each of the following categories: serious mental illnesses (SMI; ICD-10 codes F06.0, F06.2, F20.0–F20.9, F22.–F31.9, F53.), major depressive disorders (MDD; F32.0–F32.5, F32.9, F33.0–F33.42, F33.9), post-traumatic stress disorder (PTSD; F43.10–F43.12), anxiety disorders (AD; F06.4, F40.00–F40.298, F40.8, F40.9, F41.1, F41.3, F41,8, F41,9, F42., F42.2–F42.4, F42.8, F42.9, F45.20, F45.21, F45.29), and non-tobacco substance use disorders (SUD; F10–F16, F18–F19) [34].

### 2.2. Data Analytic Plan

We used multinomial logistic regression to test whether the odds of being a daily or non-daily tobacco user varied over time, by demographic group, or with the presence of specific psychiatric diagnoses. This method is very similar to the one used in many previous studies [35,36,37,38]. Multinomial logistic regression was chosen for use here because it allows for an outcome variable with 3 or more levels in which a reference or baseline level of the outcome is chosen and compared individually to each other level. In this case, the outcome variable was tobacco use status. Non-user was chosen as the reference level, and thus analyses compared non-users first to non-daily and then to daily users. For the analysis of differences between time periods, time was dummy-coded such that the period from 19 March 2020 to 15 November 2020 (early COVID) was the reference category to which pre-COVID (1 November 2019–18 March 2020) and peak COVID (16 November 2020–28 February 2021) periods were compared. To evaluate whether the diagnostic and/or demographic predictors might moderate changes over time, the initial model included interactions between linear time and each demographic (age, sex, race/ethnicity) and diagnostic (SMI, MDD, AD, PTSD, SUD) variable. For the race/ethnicity predictor, the group identifying as White was used as the reference group. Non-significant interactions were omitted, and the model was refit. For interactions that were significant or appeared to be of interest, follow-up analyses were performed by evaluating the model separately for each level of the moderating variable (e.g., for men and women separately). Analyses were conducted in Intercooled Stata 15.1 (StatCorp LLC, College Station, TX, USA). P-values less than 0.05 were considered statistically significant.

## 3. Results

Descriptive statistics for each time period are shown in Table 1. Bivariate analyses indicated that there were significant differences across time periods in terms of the prevalence of tobacco use, sex, PTSD, and SUD.

The primary model (Table 2) evaluated predictors of tobacco use status and interactions with time. In the comparison of non-users to non-daily users, there was a significant time * age interaction, indicating that the association between age and tobacco use status varied with time. There was a trend toward a time * sex interaction predicting both non-daily and daily use compared with non-use. There was also a significant time * age interaction for non-daily use but not for daily use. Notably, because these interactions were retained in the model, the main effects of both age and sex reflect the predicted associations when both variables = 0 (i.e., for male participants at the mean age). These main effects indicate that the likelihood of being a non-daily tobacco user for such individuals declined 21% from pre- to early COVID and declined 26% from early to peak COVID. In contrast, the likelihood of being a daily user rather than a non-user did not change from pre- to early COVID but declined 14% from early to peak COVID. Older male Veterans were less likely than younger Veterans to be both non-daily and daily tobacco users.

None of the psychiatric diagnosis variables interacted with time, meaning that associations with tobacco use status did not change over time. Having an active SUD diagnosis was associated with 133% greater risk of being a non-daily user than a non-user, but no other diagnostic variables predicted non-daily use. Active SUD diagnosis predicted 300% greater likelihood of daily versus non-use. Similarly, those with active SMI diagnoses were 116% more likely to be daily versus non-users compared to those without SMI diagnoses, and having an active MDD diagnosis predicted 8% greater risk of daily use compared with non-use. In contrast, having an anxiety disorder was associated with 26% lower odds of daily use compared with non-use. Non-daily use was also 31% more likely among those identifying as Black, and 20% more likely among those identifying as Asian, compared with those who identified as White. Similarly, daily use was 37% less likely among those who identified as Asian. The risk of daily use versus non-use did not otherwise differ by race/ethnicity.

**Figure 1:**
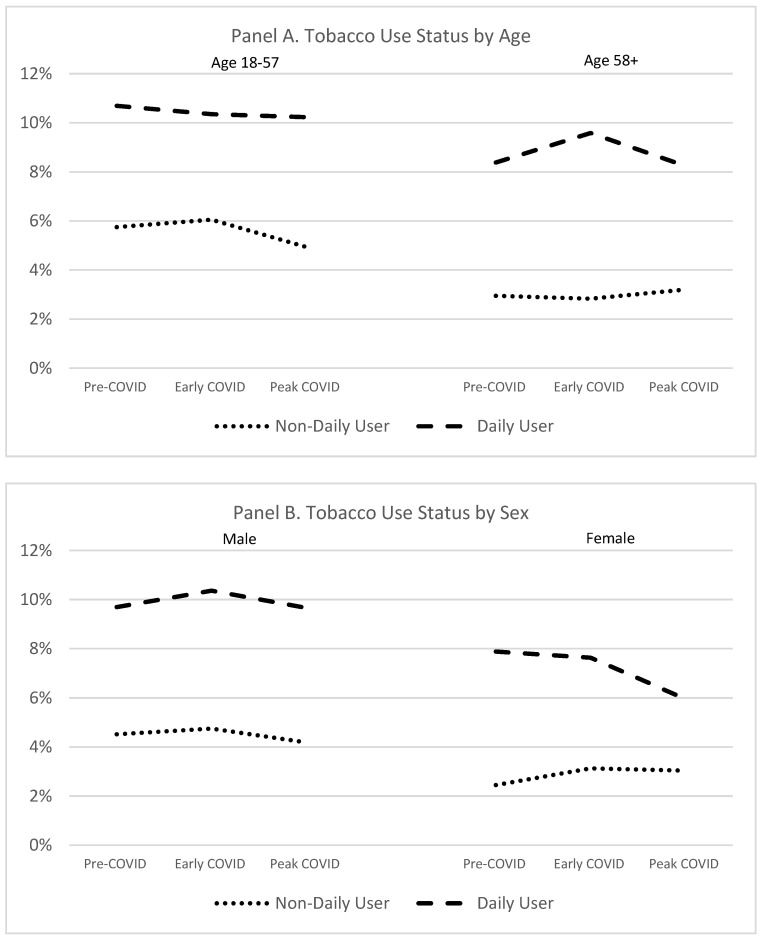
Tobacco use status over time for younger versus older (**Panel A**) and for male versus female (**Panel B**) Veterans.

The primary model suggested that age and possibly sex predicted tobacco use status differentially over time. To better understand these interactions, we conducted additional tests to examine tobacco use status over time (Figure 1). First, we performed a median split on age and refit the original model separately for both halves of the sample. Among those aged 18–57, the likelihood of being a daily user during the early COVID period did not differ from either the pre-COVID (relative risk ratio (RRR) = 1.02 (95% confidence interval 0.93, 1.11)) or peak COVID (RRR = 0.97 (0.88, 1.07)) periods. The likelihood of being a non-daily user did not change from pre- to early COVID (RRR = 0.97 (0.87, 1.09)) but did decline 23% during peak COVID compared to early COVID for this younger half of the sample (RRR = 0.77 (0.68, 0.88)). In contrast, for the older half of the sample, the likelihood of non-daily use did not change over time (pre- to early COVID: RRR = 1.04 (0.89, 1.21); early to peak COVID: RRR = 1.13 (0.95, 1.34)). However, the likelihood that Veterans aged 58 and up would be daily tobacco users was 12% lower during the pre-COVID versus early COVID time frame (RRR = 0.88 (0.81, 0.97), and then declined by 14% from early to peak COVID (RRR = 0.86 (0.77, 0.95)). Taken together, these data demonstrate that younger Veterans showed no significant change in tobacco use from the pre- to early COVID periods but were less likely to be non-daily tobacco users during the peak than the early period of the pandemic, while older Veterans had an increase in daily tobacco use from the pre- to the early pandemic periods that reversed during the peak period of the pandemic.

Next, we fit the model separately for men and women. Among men, these analyses showed that the likelihood of non-daily versus non-use was 23% higher during the pre-COVID than the early COVID period (RRR = 1.23 (1.02, 1.49)), and then declined by 27% from early to peak COVID (RRR = 0.73 (0.60, 0.89)). In contrast, the likelihood of being a daily tobacco user did not change from pre- to early COVID (RRR = 1.02 (0.89, 1.18)) or from early to peak COVID (RRR = 0.88 (0.76, 1.02)). The model that included only women indicated that the likelihood of being a non-daily tobacco user did not change from either pre- to early COVID (RRR = 0.67 (0.34, 1.30)) or from early to peak COVID (RRR = 1.18 (0.62, 2.24)). Similarly, the likelihood of daily use was not significantly different during pre- versus early COVID (RRR = 1.41 (0.94, 2.12)). However, women were 46% less likely to be daily users during the peak compared with the early COVID periods (RRR = 0.54 (0.35, 0.85)).

## 4. Discussion

Study findings demonstrate that the prevalence of non-daily tobacco use declined from the early to peak stages of the COVID-19 pandemic among younger Veterans but not among their older peers (whose non-daily tobacco use stayed roughly the same). Daily tobacco use among older Veterans increased between the pre- and early COVID periods, but then declined between the early and peak periods. Furthermore, the study results gave an indication that women have a greater reduction in daily tobacco use between the early and peak stages of the COVID-19 pandemic than men. Individuals diagnosed with serious mental illness or substance use disorders were more likely to report daily tobacco use overall during all study periods while individuals diagnosed with anxiety disorders were less likely to report daily tobacco use overall during all study periods, but psychiatric diagnoses did not predict differential change in prevalence of either daily or non-daily use. Asian or Latinx veterans were less likely to report current tobacco use during all study periods.

As for the lower prevalence of non-daily tobacco use by younger Veterans during the peak of the COVID-19 pandemic, this finding is consistent with a study from the early pandemic lockdown in Italy, where younger and occasional users were more likely to report reductions in tobacco use [39]. This finding could be explained by the social role of tobacco use among youth that ended with the social isolation due to lockdown (made worse by the peak of the pandemic in San Diego) and with the higher likelihood of successful quitting attempts among youth compared with older users [39]. Fear of contracting the coronavirus has also been shown to decrease tobacco use behavior [40], which may have been more prevalent among younger non-daily tobacco users. The majority of tobacco users believe that such use places them at greater risk of serious complications from COVID-19 [18]. A study of adults in the UK in the same age range as the younger group here found that lockdown enables quitting through lifting social barriers and allowing for a focus on health benefits [41], which supports the above explanations for the findings here. As for the finding in older tobacco users, prior research demonstrates that the early period of the COVID-19 pandemic resulted in increased social isolation for individuals over the age of 60 [42]. Increased social isolation can have a detrimental effect on mental and physical health, which could explain why tobacco use among older Veterans increased during the early COVID period.

The suggestion here of possible differences in daily tobacco use between men and women can potentially be explained by gender behavior and lifestyle choices. Women may be more likely to reduce tobacco use due to a greater concern about COVID-19 risk. A recent study that was conducted in Spain suggested that women have a ’more responsible’ attitude toward the COVID-19 pandemic than men do [43]. This may in turn have an impact on behaviors, such as tobacco use, which would explain the differences seen in the results here. Other behaviors, like mask wearing, have followed a similar trend where in one study women were observed as 1.5 times more likely than men to wear a mask while in public [44]. This discrepancy in behavior between genders could also explain why men are more vulnerable to COVID-19 than women [45], although there are some known genetic [46] and immunologic [47] risk factors at play as well.

Overall differences were found throughout, though not between, the study periods according to some psychiatric and substance use diagnoses. The increased prevalence of daily and non-daily tobacco use among those with SMI and substance use disorders is in accordance with existing studies that show tobacco use being more prevalent among people with psychiatric illness than the general population [48]. Perhaps MDD and PTSD diagnoses were not associated with daily tobacco use, and anxiety disorders were actually associated with decreased use because this is in relation to the rest of the population in the study. Our sample is relatively high in overall mental health severity, with 36% having at least one mental health diagnosis, and includes those with multiple mental health diagnoses (23% had at least 2 diagnoses), SUD, or other SMI, the latter two proven to be very strong predictors of nicotine dependence [49,50]. Additionally, the definition of anxiety disorders in the VA system used in this study includes disorders which are potentially less severe, such as social anxiety and phobias as well as obsessive-compulsive disorder, known to have the lowest prevalence of tobacco use among the anxiety disorders [51]. The examination of tobacco use prevalence in times of stress in people with mental illness is particularly important because sensory perception plays an important role in emotional processes, and traumatic experiences can lead to extreme sensory processing patterns which can have a significant effect on an individual’s quality of life [52].

The overall findings of decreased daily tobacco use among the Asian and Latinx Veterans but increased non-daily use among the Black and Latinx Veterans, compared to White Veterans, is also in line with previously published reports that racial/ethnic minorities have higher rates of light and intermittent tobacco use, but generally lower overall tobacco use prevalence rates than non-Hispanic Whites [53]. We speculate that part of this finding may represent a shift in Latinx tobacco use from daily to non-daily use. This shift in frequency could be explained by the disproportionate number of U.S. COVID-19 cases in Latinx communities during the early COVID period [54], which may have heightened concern about the risk of tobacco use and changed use behavior. This speculation is supported by recent studies that have shown significantly higher rates of COVID-19 fear [55] and psychological distress [56] in Latinx populations.

A limitation of the study was that it utilized already existing electronic medical record data of Veterans receiving VA care, which may not be representative of the general population, due to the potential risk of informed presence bias [57]. A patient’s presence in an EMR database was not random; rather, it signified that the patient had a need for a healthcare visit [58]. Access to healthcare also could have changed over the time periods of the pandemic (pre-, early, and peak COVID-19), which may have influenced the profile of patients being seen [59,60]; however, healthcare providers at the VA San Diego remained accessible to patients due to the availability of both telephone and video visits. Another limitation of the study was that the use of existing data assumed that components of each visit were relatively uniform and did not affect answers to the standard tobacco use status question stated above (Materials and Methods, paragraph 1). The data collected were additionally limited to the San Diego area and may not be applicable to other parts of the country, though San Diego has a somewhat diverse population in terms of race/ethnicity [61]. Another limitation was that the study used existing chart data without obtaining participant information regarding motives for changing their tobacco use patterns, and whether they were COVID-related. This limitation prevents us from drawing firm conclusions about why participants’ tobacco use patterns changed during the peak of the pandemic (as was done in survey studies during the early pandemic [9,10,11,12,13,14,15,16,17,18,19,20,21,22,23,24,25]).

The use of EMR-based data to conduct research has both pros and cons. They can provide researchers with substantial health data for geographically, socioeconomically, and culturally diverse populations and they are also cost-efficient and require considerably less time to complete than studies using primary data collection methods [62].

While EMR-based data has known limitations, some were minimized by our study methods while a few remain as limitations. The use of the EMR-based data set here from the VA (which is a closed health system with uniform questions) addresses several areas identified as challenges for EMR research [63,64], namely that a standard EMR function was used, structured data was collected, and all data within the VASDHS (including across sites) was accessible. Other issues identified as challenges [63] are limitations of this study, namely the absence of a customized set of questions (e.g., items from the Fagerström Test for Nicotine Dependence or other standard rating scales [65]), lack of all patients seen at the VA having the smoking assessment filled out [65], and potential changes in EMR reliability over time [64]. A known issue with multisite EMR-based research is that it can amplify challenges of using EMR data, such as cross-institutional differences in data capture and workflows, use of standards, and missingness [66]. The use of a single system/site here lessened the risk of problems inherent to multisite research (such as site differences in data collection and storage [67]), though the use of a single system/site here could potentially limit generalizability.

Previous research has shown the importance of active solicitation of information [68] and EMRs are now being used to help evaluate the effectiveness [69] and potential side effects [70,71] of COVID-19 vaccines. Similarly, EMR-based data has been utilized previously in studies looking at the effects of the COVID-19 pandemic as a stressor on people with bipolar disorder [72], the safety of the COVID-19 vaccine in pregnant women [73], the effect of COVID-19 on pregnancy outcomes [74], the effects of clinical characteristics on outcomes of COVID-19 patients [75,76,77], the factors that influence pharmacotherapy for tobacco dependence in the VA system [78], the effect of mental health disorders on COVID-19 mortality [79], COVID-19 vaccine adverse effects [68], and the effects of the COVID-19 pandemic on body mass index in children [80]. Thus, the present study builds upon a growing body of research examining similar effects of the COVID-19 pandemic.

## 5. Conclusions

This study found that the overall likelihood of daily and non-daily tobacco use did not differ between the pre-, early, and peak COVID-19 periods, but that differences existed for some demographic groups. Specifically, the odds of being a non-daily user was lower in the peak COVID-19 period than in the early COVID-19 period for younger Veterans but not their older counterparts. In addition, older Veterans had a higher prevalence of daily tobacco use during the early COVID-19 period that normalized during the peak COVID period. The study results also gave an indication that the rate of daily use decreased by a greater amount between the early and peak periods in female than male Veterans. The differences in tobacco use behavior among female and male Veterans encourages further research on the topic. These findings demonstrate factors that may affect willingness to decrease tobacco use during the time of a severe public health crisis. The central practical implication of this study is that specific tobacco-using subgroups (such as older non-daily tobacco users and male daily tobacco users) may need targeted interventions to increase participation in tobacco use cessation programs during times of increased stress. These subgroups could be the focus of extra attention because they did not decrease tobacco usage during the peak of the pandemic, as did other subgroups. Greater use of motivational interviewing [81] could be employed, which may result in substantial benefits to their health.

## Figures and Tables

**Table 1:** Descriptive statistics for key variables during the pre-, early, and peak COVID periods.

Variable	Pre-COVID (1 November 2019–18 March 2020)	Early COVID (19 March 2020–15 November 2020)	Peak COVID (16 November 2020–28 February 2021)
Visits (61,852)	21,809	25,233	14,810
Age—mean (SD)	57.4 (17.3)	55.2 (17.5)	56.6 (17.4)
Sex—% male	87.8%	86.4%	87.5%
Racial/ethnic group—% white	55.2%	53.3%	55.3%
Daily tobacco user	9.5%	10.0%	9.2%
Non-daily tobacco user	4.3%	4.5%	4.2%
SMI	4.2%	4.6%	4.8%
MDD	12.9%	13.1%	13.3%
PTSD	19.1%	20.3%	19.8%
AD	9.3%	9.7%	9.1%
SUD	8.1%	9.0%	8.9%

Legend: SMI = serious mental illness; MDD = major depressive disorder; PTSD = post-traumatic stress disorder; AD = anxiety disorder; SUD = substance use disorder.

**Table 2:** Multinomial logistic regression model predicting tobacco use status for pre-COVID and peak COVID compared with early COVID.

Predictor	Subgroup	Relative Risk Ratio (RRR)	95% c.i.	Std. Err.
Intercept		0.23	0.21, 0.26	0.01
	Non-Daily Tobacco Use			
Time	Pre-COVID	1.21	1.00, 1.45	0.11
	Peak	0.74	0.61, 0.90	0.07
Race/ethnicity	Black	1.31	1.19, 1.46	0.07
	Asian	0.80	0.69, 0.92	0.06
	Latinx	1.12	0.93, 1.34	0.11
	Other	1.07	0.95, 1.22	0.07
Sex		0.31	0.21, 0.46	0.06
Time × Sex		1.21	1.00, 1.46	0.12
Age		0.97	0.96, 0.97	0.01
Time × Age		1.00	1.00, 1.01	0.01
SMI		1.86	0.87, 3.94	0.71
MDD		0.95	0.84, 1.07	0.06
AD		0.98	0.87, 1.12	0.06
PTSD		1.08	0.98, 1.19	0.05
SUD		2.33	2.08, 2.61	0.13
	Daily Tobacco Use			
Time	Pre-COVID	1.04	0.90, 1.19	0.07
	Peak	0.86	0.74, 0.99	0.06
Race/ethnicity	Black	1.02	0.95, 1.10	0.04
	Asian	0.63	0.57, 0.70	0.03
	Latinx	0.97	0.85, 1.10	0.07
	Other	0.53	0.47, 0.59	0.03
Sex		0.79	0.61, 1.01	0.10
Time × Sex		0.89	0.79, 1.01	0.06
Age		0.98	0.98, 0.99	0.01
Time × Age		1.00	1.00, 1.00	0.01
SMI		2.16	1.31, 3.55	0.55
MDD		1.09	1.00, 1.18	0.04
AD		0.74	0.67, 0.82	0.04
PTSD		1.03	0.96, 1.10	0.04
SUD		4.00	3.72, 4.30	0.15

Legend: The early COVID period was the reference category for time, and White race was the reference category for race/ethnicity. Sex was coded as 0 = male, 1 = female. Age was mean centered at 0. c.i. = confidence interval; Std. Err. = standard error; SMI = serious mental illness; MDD = major depressive disorder; AD = anxiety disorder; PTSD = post-traumatic stress disorder; SUD = Substance Use Disorder.

## Data Availability

The data are not publicly available. A de-identified data set can be obtained by contacting the authors.
